# Supplementary figures and images for: Sequence characterization of eccDNA content in glyphosate sensitive and resistant Palmer amaranth from geographically distant populations
Source: PLoS One. 2022 Sep 14;17(9):e0260906. doi: 10.1371/journal.pone.0260906 (PMC9473621; doi:10.1371/journal.pone.0260906)

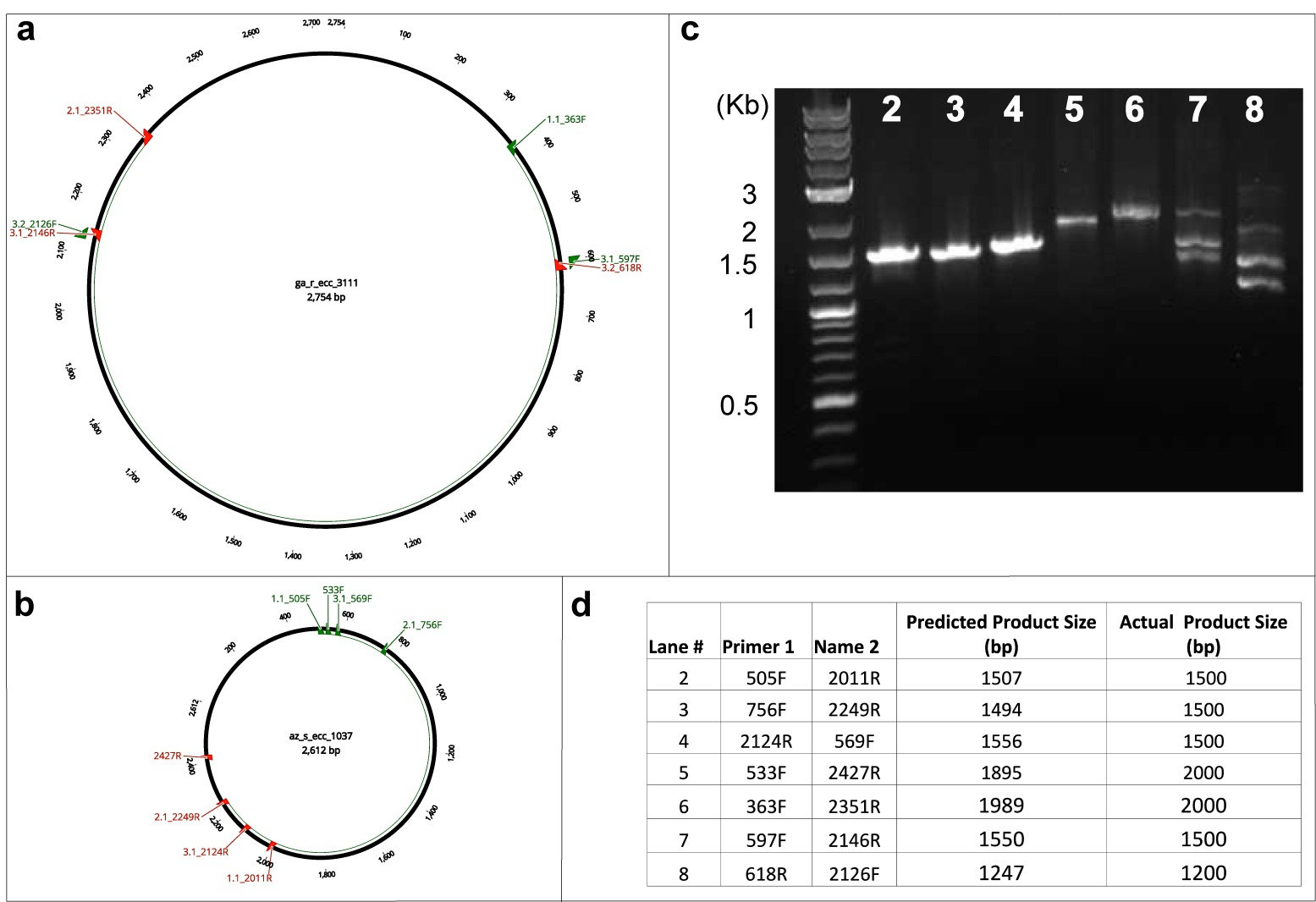

Supplement: S1 Fig — A./B. candidate eccDNA from Georgia resistant and Arizona sensitive biotypes with locations of primers. C. Agarose gel with amplicons D. Table of primers used, predicted and observed PCR amplicon sizes. (TIF) [file pone.0260906.s011.tif]
